# Can You Judge a Disease Host by the Company It Keeps? Predicting Disease Hosts and Their Relative Importance: A Case Study for Leishmaniasis

**DOI:** 10.1371/journal.pntd.0005004

**Published:** 2016-10-07

**Authors:** Christopher R. Stephens, Constantino González-Salazar, Víctor Sánchez-Cordero, Ingeborg Becker, Eduardo Rebollar-Tellez, Ángel Rodríguez-Moreno, Miriam Berzunza-Cruz, Cristina Domingo Balcells, Gabriel Gutiérrez-Granados, Mircea Hidalgo-Mihart, Carlos N. Ibarra-Cerdeña, Martha Pilar Ibarra López, Luis Ignacio Iñiguez Dávalos, María Magdalena Ramírez Martínez

**Affiliations:** 1 Instituto de Ciencias Nucleares, Universidad Nacional Autónoma de México, Ciudad de Mexico, Mexico; 2 C3 - Centro de Ciencias de la Complejidad, Universidad Nacional Autónoma de México, Ciudad de Mexico, Mexico; 3 Instituto de Biología, Universidad Nacional Autónoma de México, Ciudad de Mexico, Mexico; 4 Unidad de Investigación en Medicina Experimental, Facultad de Medicina, Universidad Nacional Autónoma de México, Ciudad de Mexico, Mexico; 5 Laboratorio de Entomología Médica, Departamento de Zoología de Invertebrados, Facultad de Ciencias Biológicas, Universidad Autónoma de Nuevo León, Nuevo León, México; 6 Facultad de Estudios Superiores-Zaragoza, Universidad Nacional Autónoma de México, México D.F., México; 7 División Académica de Ciencias Biológicas. Universidad Juárez Autónoma de Tabasco, Villahermosa, Tabasco, México; 8 Departamento de Ecología Humana, Centro de Investigación y de Estudios Avanzados del Instituto Politécnico Nacional (Cinvestav) Unidad Mérida, Mérida, Yucatán, México; 9 Laboratorio de Zoología, Centro Universitario de la Costa Sur, Universidad de Guadalajara, Jalisco, México; 10 Departamento de Salud y Ecología Humana, Centro Universitario de la Costa Sur, Universidad de Guadalajara, Jalisco, México; University of Liverpool, UNITED KINGDOM

## Abstract

Zoonoses are an important class of infectious diseases. An important element determining the impact of a zoonosis on domestic animal and human health is host range. Although for particular zoonoses some host species have been identified, until recently there have been no methods to predict those species most likely to be hosts or their relative importance. Complex inference networks infer potential biotic interactions between species using their degree of geographic co-occurrence, and have been posited as a potential tool for predicting disease hosts. Here we present the results of an interdisciplinary, empirical study to validate a model based on such networks for predicting hosts of *Leishmania (L*.*) mexicana* in Mexico. Using systematic sampling to validate the model predictions we identified 22 new species of host (34% of all species collected) with the probability to be a host strongly dependent on the probability of co-occurrence of vector and host. The results confirm that *Leishmania* (*L*.) *mexicana* is a generalist parasite but with a much wider host range than was previously thought. These results substantially change the geographic risk profile for Leishmaniasis and provide insights for the design of more efficient surveillance measures and a better understanding of potential dispersal scenarios.

## Author Summary

Emerging and neglected zoonoses are an important global threat to public health. Host range, in particular, is a crucial factor in determining disease risk and the potential for adequate interventions. Here we show that *Leishmania* has a very wide host range and that Complex Inference Networks can be used to infer ecological relationships in the context of zoonoses, identifying both the potential hosts and their relative importance. These results substantially change the risk profile and potential control measures that can be used to combat the disease, allowing for the design of more efficient surveillance measures and a better understanding of potential dispersal scenarios.

## Introduction

Zoonoses are an important class of neglected [1,2] or emerging infectious diseases [3–6], accounting for more than 60% of human infectious diseases. Wildlife species that are hosts for pathogens play a fundamental role in zoonoses, threatening domestic animal and human health and global biodiversity. Although for particular zoonoses some hosts have been identified [7–14], there have been few systematic empirical studies carried out to identify the host range and the relative importance of the different hosts within that range for a given zoonosis.

Additionally, most work on disease ecology over the last 20 years has focused on single-host, single-agent systems. Recently however, there has been increasing interest in the more complex case of multi-host systems [15–20], with the realization that many zoonoses have potentially ample host ranges.

The relative importance of a species as a disease host will be highly multi-factorial, with risk factors covering many different scales, from the micro to the macro. However, there are two particularly important elements that come into play: host competence (the ability to transmit the parasite to a new host or vector) and the frequency of contact between host and pathogen or, in the case or vector borne diseases, host and vector [21].

Although it is intuitively clear that the “relative importance” of a host will depend on both its competence and its frequency of contact with the vector, it is a somewhat ambiguous concept in that it depends, for instance, on whether we are talking about human transmission or of maintaining an enzootic transmission cycle. We will take here relative importance to be associated with the probability of infection of an individual of a potential host species. Abstractly, this is a highly multi-factorial function P(C | X_1_, X_2_,…, X_N_), where, for instance, X_1_ could represent host competence and X_2_ frequency of host-vector contact. Although a host may be highly competent, if it only has infrequent contact with any disease vector, then the frequency of infected individuals will be low. Conversely, a host and a vector may have frequent contact, but the host may have low competence. All else being equal, the most important hosts will be those that have frequent contact with the disease vectors and are competent. Unfortunately, gathering information about these two aspects, especially for emerging or neglected diseases, is difficult and resource intensive [7,8,22,23]. Furthermore, how these fundamental aspects interact in multi-host systems is quite distinct from their single-host analogues. For instance, the fact that host-pathogen competencies may differ greatly among the hosts can potentially lead to a dilution effect [24–26].

Another important differentiating factor is that, multiple-host systems provide for much richer and complex scenarios for the dispersion of a disease from one geographic region to another [27]. As the characteristics of the host range play a crucial role in the emergence risk of a novel human pathogen and of the optimal interventions for combating the zoonosis [16] the importance of predicting and identifying potential disease hosts has been widely recognized [28–30]. To do so by exhaustive, systematic search through all possible hosts would be prohibitively resource intensive. At the same time, good data often only exist for a few (presumed) focal species. As it is unknown what part of the host range has already been discovered, the undiscovered part constitutes a type of ‘epidemiological dark matter’ [31].

An early attempt at systematising the search for potential hosts [32], in the case of Ebola, considered a heuristic approach based on expert knowledge, which was used to then filter the list of potential candidates. As such, it is both subjective and subject to model bias. More recently, other methods have appeared: In [33] a small group of four suspected hosts was used as a starting point for including biotic effects indirectly by calculating the fundamental niche of these four mammal species and considering the geographical correspondence with the niche distributions of the vectors. This paper was more concerned with including information about a particular set of potential hosts into corresponding risk maps rather than identifying new hosts per se. In contrast, in [34], a classification model using a supervised learning technique was used to predict other potential rodent reservoirs based on the predictive value of a set of potentially distinguishing characteristics of already known ones. Note that this paper was concerned with the potential hosts of a large number of pathogens considered all together and therefore could not discriminate against potential hosts of one disease versus another. In this case only categorical and no spatial information was used. Moreover, as it is based on supervised learning it can be affected by bias in the data defining both the class and in the predictors. This is in evidence in that the most predictive factor found was the number of literature citations for a given species. In [35], the authors considered biotic factors as potential predictive variables for describing the geographic range of Ebola rather than trying to predict which mammals are the most likely hosts.

In contrast, in Stephens et al [36], a general framework was presented using Complex Inference Networks based on the degree of co-occurrence between different species, for inferring potential biotic interactions. The framework is also capable of including in other variable types, at distinct spatial resolutions, such as environmental layers normally associated with abiotic variables [37] allowing for a comparison of the relative importance of biotic versus abiotic factors.

The methodology differs from those of [32–35] by using as model inputs only purely spatial data, using point collection data to proxy spatial distributions of taxa and co-occurrences to infer potential biotic interactions. In particular, it uses no auxiliary information, such as expert knowledge, as in the case of [32]; fundamental niche distributions of taxa, as in the case of [33]; or specific categorical data associated with the relevant taxa, as in the case of [34].

Networks are an important tool in ecological studies [38–41]. However, their local structure—in the sense of two nodes and a link as the base element—represents an already known relation, such as in a food web [38], or in a contact network representing ticks, vertebrates and pathogens, as in [42]. In this case the local structure of the network, i.e., the individual nodes and links, only represent what is known. However, the global properties of the network can lead to new insights from an eco-systemic or community viewpoint and also to specific predictions. In contrast, Stephens et al. [36] use the local structure of networks to infer and discover previously unknown relations, such as the relation between vector and host. Although nodes are taxa, the local structure of the network is different to a traditional ecological network in that the links represent the degree of overlap between the distributions of the corresponding taxa with the idea that statistically significant degrees of co-occurrence can be an indicator of potential biotic interaction between the associated taxa, such as between a host and a vector.

In determining the host range of a zoonosis, an exhaustive empirical analysis of all potential hosts is prohibitively difficult, hence the importance of theoretical models, such as that presented in [36], for guiding observation and experiment. Although consistent with known results, it is important to note that the theoretical predictions of [36] have not previously been tested experimentally. A theoretical model needs to be validated by experiment, as this is the only way to truly determine if the model works. In this vein, most ecological modelling of zoonoses remains untested, in that theoretical predictions are not validated using a suitable experimental validation framework. This paper presents the results of an interdisciplinary study carried out to experimentally test the predictions of [36] using, as a test, the case of Leishmaniasis in Mexico.

Leishmaniasis is a significant, yet neglected tropical disease, with 350 million people in 98 countries worldwide living at risk of developing one of the many forms of the disease [43]. It is caused by infection with one of several different species of protozoan parasites of the genus *Leishmania*, which maintain their life cycle through transmission between an insect (sandflies—genus *Lutzomyia*) and a mammalian host. In Mexico, the most epidemiologically important species is *Leishmania* (*L*.) *mexicana*, though the presence of other species has been confirmed. Eleven species of *Lutzomyia* are considered to have potential medical importance. Of these, three are known vectors of either cutaneous or visceral Leishmaniasis, while four others have been found infected with *L*. (*L*.) *mexicana* [44].

Besides the clinical and social importance of Leishmaniasis [45] and the acknowledgment of its zoonotic nature [46], the identification of wildlife hosts for these parasites is sparse and non-systematic. Prior to the present study, only 8 mammalian species had been identified as hosts in Mexico [47–49]. This potential lack of knowledge of parasite hosts greatly increases the difficulty of formulating theoretical approaches to explaining and predicting disease spread or for planning better and sustainable control measures [50].

## Methods

### Ethics statement

The collection of specimens was performed according to the guidelines of the American Society for the Use of Mammalogists of Wild Mammals in Research and under a collecting permit is- sued by the General Direction of Wildlife of Mexico (permission number SGPA/DGVS/04631/ 14). The infections in mice were carried out following the National Ethical Guidelines for laboratory animals NOM-062-ZOO-1999. The project was approved by the Institutional Ethics Committee of the Medical Faculty of the National Autonomous University of Mexico (UNAM) with the registration number FMED/CI/RGG/013/01/2008.

### Rationale for the modelling methodology

The general modelling methodology of [36], is based on the idea that biotic interactions can be inferred from the locations of taxa as a function of space and time. Although biotic and ecological interactions in general are very complex, it is reasonable to state that the spatio-temporal distributions of taxa, or other ecological variables, reflect all of the factors and their causal interactions that determine them. In [36], the degree of co-occurrence between taxa was taken as an observable measure with which potential interactions could be inferred. Although co-occurrence is not equal to biological interaction, a significantly non-random co-occurrence distribution is a *necessary* condition for a biotic interaction between taxa, and as such it can be used to formulate hypotheses that can be checked experimentally. However, it is clearly not a *sufficient* condition. In the spirit of niche modelling, a biotic variable that co-occurs with a target taxon can be understood as being a niche component in the same sense as any abiotic variable, such as temperature. In fact, one would generally expect a closer causal relation between biotic variables than with abiotic variables. For example, the distribution of prey species for a predator, such as a carnivore, should clearly influence the latter’s distribution more significantly than temperature or precipitation.

In the case of many zoonoses, the predominant interaction between vector and host is due to the former feeding on the latter. This obviously requires a coincidence in space and time. Species that offer blood meals can maintain the presence of vector populations independently of the capacity to harbour a given pathogen. In other words, the interaction between host and vector is a necessary but not sufficient condition for the transmission of the pathogen. The total number of encounters between vector and potential host depends on many factors, including the abundance of both species. However, a key factor is the geographical overlap between them, as the greater the overlap the greater the probability of an encounter. Thus, for two host species, identical in all respects except their relative geographical overlap with the vectors, the host species with the larger overlap will be epidemiologically more important. Thus, vector-mammal geographical overlap is a necessary but not sufficient condition for both a feeding interaction and a pathogen transmission interaction.

Of course, there may be geographical overlap between species due to other reasons than a direct biotic interaction. Even if species distributions were random there would be overlap. It is therefore necessary to measure overlap relative to a null hypothesis, such as that associated with random distributions. Additionally, it may occur that there is a non-random overlap due to the existence of one or more confounding factors; for example, an abiotic variable, such as temperature. This can only be quantified by controlling for the presence of such a factor. As it is obviously infeasible to control systematically for every potential factor, a logic must be presented for considering a particular candidate. In summary: although geographical overlap is not a sufficient condition for biological interaction, it is necessary, and as such can be used to construct models that can then be checked explicitly by experiment to see to what extent it is predictive.

The explicit example considered in [36] was the identification of potential hosts for Leishmaniasis by studying co-occurrences between the vector species and the potential host species. A Complex Inference Network summarising the co-occurrence distributions was deduced that showed the most important potential mammal hosts for each sandfly species. Although the full network contains a great deal of structure and information, in terms of experimental validation each network observable requires an experimental protocol to be able to measure it. In particular, to work at the species level for the vectors, and associate and confirm hosts for a given vector species, would require collecting sandflies and genotyping their blood meals, as well as collecting potential host species and confirming the presence of the pathogen. In the present experimental study, we restricted attention to only potential host species and tested them for the presence of the pathogen considering the vector at the genus level only.

### Inferring vector-hosts interactions

The explicit model for predicting potential hosts was created using a database of point collections for one Class, Mammalia, and one genus, *Lutzomyia*, of the class Insecta. The mammal data set contains 37,297 unique point collections from geo-referenced localities for 419 terrestrial mammals occurring in Mexico—the full potential host range (GBIF; www.gbif.org, and CONABIO; www.conabio.gob.mx). For *Lutzomyia*, there were 270 collections points taken from published literature and from national collections: Instituto de Diagnóstico y Referencia Epidemiológica (InDRE, Mexico City), the Colección Entomológica Regional, Universidad Autónoma de Yucatán (UADY, Mérida) and the Laboratorio de Medicina Tropical at the Universidad Nacional Autónoma de México (UNAM, Mexico City).

First, we divide up a geographic region of interest into spatial cells, x_α_,–in the present case Mexico–here we used a uniform grid of 3,337 rectangular cells of size 25km x 25km. The choice of an appropriate cell size is known in geography as the “modifiable areal unit problem”. In terms of forming a spatial grid, there are at least two important considerations: The sizes of the statistical samples of the variables and their degree of correlation. Too fine a grid and there will be no co-occurrences, too rough and there will be little to no discrimination. It was checked explicitly in [36] that the relative ranking of mammals by the model was quite insensitive to the cell size over the range 5km to 100km. See also [51]. One then counts co-occurrences in each spatial cell between different taxa, or other variables. In the present case, the co-occurrences are between the presence of Lutzomyia, B_i_, and the presence of each distinct mammal species, I_k_.

We take the taxon distribution, B_i_ (*Lutzomyia*), and a subset of potential niche variables. We are interested in the probability P(B_i_ | **I**′) = NB_iAND I′_ /N_I′_, where N_BiAND I′_ is the number of spatial cells where there is a co-occurrence of the taxon B_i_ and the niche variables **I**′, which we take here to be biotic variables, and N_I′_ is the number of cells where the niche variables take their stated values. The niche profile **I**′(x_α_) associated with a spatial cell x_α_ then determines the probability of the distribution variable, B_i_(x_α_), in that cell, and one now has a predictive model. The problem of calculating P(B_i_ | **I**’) directly is that both N_Bi AND I′_ and N_I′_ are likely to be zero when the number of taxa or niche variables considered simultaneously is large, as there will tend to be no co-occurrences of so many variables. This can be ameliorated by considering a reduced number of both class and feature variables. For instance, P(B_i_ | **I**_k_) is determined by the number of co-occurrences of the taxon Bi and the particular niche variable I_k_ and, in principle, allows us to find the most important statistical associations between the niche variables and the taxa distributions. However, P(B_i_ | **I**_k_) being a probability does not account for sample size. For example, if P(B_i_ | **I**_k_) = 1, this may be as a result of there being a coincidence of B_i_ and I_k_ in one spatial cell or 1,000. Obviously, the latter is more statistically significant. To remedy this we consider the following test statistic

$$\varepsilon (B_{i}|I_{k})=\frac{N_{I_{k}}(P(B_{i}|I_{k})-P(B_{i}))}{{\left(N_{I_{k}}P(B_{i})(1-P(B_{i}))\right)}^{1/2}}$$ (1)

a binomial test which measures the statistical dependence of B_i_ on **I**_k_ relative to the null hypothesis that the distribution of B_i_ is independent of **I**_k_ and randomly distributed over the grid, i.e., P(B_i_) = $N_{B_{i}}/N$, where $N_{B_{i}}$ is the number of grid cells with point collections of species B_i_ and N is the total number of cells in the grid. The sampling distribution of the null hypothesis is a binomial distribution where, in this case, every cell is given a probability P(B_i_) of having a point collection of B_i_. The numerator of eq (1) then, is the difference between the actual number of co-occurrences of B_i_ and I_k_ relative to the expected number if the distribution of point collections were obtained from a binomial with sampling probability P(B_i_). As we are talking about a stochastic sampling the numerator must be measured in appropriate “units”. As the underlying null hypothesis is that of a binomial distribution, it is natural to measure the numerator in standard deviations of this distribution and that forms the denominator of eq (1). In general, the null hypothesis will always be associated with a binomial distribution as in each cell we are carrying out a Bernoulli trial (“coin flip”). However, the sampling probability can certainly change.

The quantitative values of ε(B_i_ |I_k_) can be interpreted in the standard sense of hypothesis testing by considering the associated p-value as the probability that |ε(B_i_ |I_k_) | is at least as large as the observed one and then comparing this p-value with a required significance level. In the case where $N_{I_{k}}$ > 5–10 then a normal approximation for the binomial distribution should be adequate, in which case ε(B_i_ |B_k_) = 1.96 would represent the standard 95% confidence interval. When a normal approximation is not accurate then other approximations to the cumulative probability distribution of the binomial must be used.

As ε increases monotonically with the frequency of co-occurrence, we interpret a statistically significant positive correlation as inferring a potential biotic interaction. Here, between sandflies and the corresponding mammal, which in this ecological setting one would naturally interpret as the mammal being a blood source for the sandfly, and therefore a potential host. The higher the value of (P(B_i_ | I_k_)—P(B_i_)) the greater the degree of spatial overlap between the species distributions and therefore the greater the risk posed by the corresponding mammal. Negative values of ε correspond to spatial overlaps that are less than one would expect from the null hypothesis.

The 419 mammal species were ranked according to ε. The resultant list serves as a predictive risk model, with the hypothesis that the highest ranked mammals correspond to the most important hosts, where, in the absence of other information, we assume that host competence is equal and importance is associated with the degree of spatial overlap between sandflies and mammal. All else being equal more overlap means more vector-host encounters. It should be noted that the method is not determining the physiological capacity of a mammal species to be a host but, rather, its potential epidemiological importance given that presence of mammal hosts is a necessary condition for the presence of the pathogen.

### Constructing risk maps from inference networks

A corresponding biotic geographic risk model can be computed by calculating the probabilities P(B_i_ |**I**′), or proxies thereof, for each spatial cell. When **I**′ is of high dimension, this can be done using different classification models, such as neural networks, discriminant analysis, etc. A particularly transparent, simple and effective approximation is the Naive Bayes approximation:

$$P(B_{i}|I)=\frac{P(I|B_{i})P(B_{i})}{P(I)}=\frac{\prod_{k=1}^{N}P(I_{k}|B_{i})P(B_{i})}{P(I)}$$ (2)

where, in the first equality, Bayes rule has been used, and in the second it has been assumed that the niche variables I_k_ are independent. The product here is over the N niche variables under consideration as conditioning factors for B_i_. In the case of the relationship between *Lutzomyias* and mammals, N represents the number of mammal species. A score function that can be used as a proxy for P(B_i_ |**I**′) is

$$S(B_{i}|I\prime )=\sum_{k=1}^{N}S(B_{i}|I_{k})=\sum_{k=1}^{N}\text{ln}\left(\frac{P(I_{k}|B_{i})}{P(I_{k}|\overset{\_\_}{B_{i}})}\right)$$ (3)

where $\bar{B_{i}}$ is the complement of the set B_i_. For example, if B_i_ is the set of cells with presence of taxon B_i_ then represents the set of cells without presence. S(B_i_ | **I**′) is a measure of the probability to find the distribution variable B_i_ when the niche profile is **I**′. It can be applied to a spatial cell x_α_ by determining the niche profile of the cell, **I**′(x_α_). As an example, for two biotic niche variables, B_2_ and B_3_, that take values 1 (corresponding to the fact that there is a point collection associated with that cell) and 0 (there is no point collection associated with the cell), the four possible biotic niche profiles of any cell are (B_2_, B_3_) = (0,0), (0,1), (1,0) and (1,1). The score contributions of each biotic variable are S(B_i_|B_2_) and S(B_i_|B_3_), calculated using the above formula. Hence, S(B_i_ | **I**′) = S(B_i_ | B_2_, B_3_) = S(B_i_|B_2_) + S(B_i_|B_3_). Thus, for any given spatial cell x_α_ one can assign a niche profile, i.e. values of B_2_ and B_3_, from whence it is possible to assign a corresponding score. If there is no statistical association between B_i_ and B_2_ or B_3_ then the corresponding score contributions are zero.

An overall zero score signifies that the probability to find B_i_ is the same as would be found if B_i_ were distributed randomly. If the score is positive then there is a higher than random probability to find B_i_ present and on the contrary if the score is negative. As each niche factor is treated separately in ε(B_i_ |I_k_) or S(B_i_|I_k_) we can thus evaluate the relative contribution of any given niche factor and compare it to the contribution of any other. By determining the set of presence/no presence attributes in a spatial cell, eq (3) can be applied to each cell thereby determining the relative risk of that cell for the presence of *Lutzomyias*. As taking the ranked list as a predictive model involves several important assumptions; it is essential to test the model with experimental data. Obtaining the relevant data required sampling the spatial grid by collecting mammals and sandflies from different geographic points and then testing them for the presence of the parasite.

### Sampling design for model testing

The sampling sites were selected as follows: a 25 x 25 km grid (as discussed above) was superimposed on a map of Mexico and this was used as template to determine the sampling sites. The sampling was stratified according to altitude so that only grid squares at < 2000 masl were used. Also, we excluded any grid square with >50% of water or urban cover. Sometimes the 25 x 25 km grid square selected was sub-divided in order to have more than one sampling site per square. Once the grid was established we selected 52 localities at random in 10 Mexican states and covering many different eco-regions and associated with a large selection of vegetation types. A random sample of spatial cells was necessary in order to fairly validate the model. If we had targeted the sampling to only those cells predicted to be highest risk then we would not be able to discriminate between high and low risk in the validation. Of course, once the model is validated it can be used with confidence in the future to preferentially identify those regions of highest risk where, for example, surveillance efforts should be targeted. The sampling was carried out by four field groups who, over a period of two years, collected 922 taxonomically identified specimens of 70 distinct species (for more details of animal sampling see S1 Text).

### Laboratory methodology

Tissue samples were taken to the Laboratorio de Inmunoparasitología of the Unidad de Investigación en Medicina Experimental of the National Autonomous University of Mexico, UNAM, where PCR tests were carried out to identify the presence of the pathogen *Leishmania (L*.*) mexicana*.

#### DNA purification

DNA from animal tissues was purified from approximately 25 mg of starting material. DNA extractions were done with a DNeasy Blood and Tissue kit (QIAGEN, Germany), following the manufacturer’s instructions. Genomic DNA was used for PCR-based amplification.

#### Oligonucleotides

To determine the presence of *Leishmania* we used oligonucleotides based on the *Leishmania* mini-circle kinetoplast DNA sequences that are conserved among species. The primer sequences used for amplification were 5´-CTRGGGGTTGGTGTAAAATAG-3´ (L.MC-1S) and 5´-TWTGAACGGGRTTTCTG-3´ (L.MC-1R) [52]. For the species *Leishmania (L*.*) mexicana* we took primer IR1, designed by [53], which corresponds to the 32 final nucleotides of the conserved sequences from the 3’ region of the small subunit of the 18S ribosomal gene as our forward primer: IR1 (5’- GCT GTA GGT GAA CCT GCA GCA GCT GGA TCA TT-3’), the reverse primer was LM17 (5′-CCC CTC TCC TCC TCC CC-3′) [54].

#### Polymerase chain reaction amplification

The PCR was performed using 50 μl of the following reaction mixture: Taq PCR Master Mix (QIAGEN) (provides a premixed solution containing Taq DNA Polymerase, PCR buffer, MgCl2 and dNTPs), 100 ng of the corresponding oligonucleotides, and DNA from tissues, we used 1 μl of tissue extract corresponding to 100 ng of DNA. The amplification was performed in a Perkin Elmer 2720 thermocycler using different conditions, which depended on the oligonucleotides used. For L.MC-1S/ L.MC-1R *Leishmania* genus we used the PCR amplification with *Leishmania* mini-circle kinetoplast DNA specific primers was performed with 30 cycles of denaturation (95°C for one minute), annealing (55°C for one minute), and polymerization (72°C for one minute). For IR1/LM17 *L*. (*L*.) *mexicana* we used 35 cycles of 1 min at 94°C, 1 min at 65°C and 1 min at 72°C. In all cases the cycles were preceded by another cycle at 94°C for 5 min and a final extension cycle of 72°C for 7 min.

An analysis of the sensitivity of the primers L.MC-1S/ L.MC-1R was made with DNA from cultured promastigotes of *L*. *(L*.*) mexicana* using: 10 ng, 1 ng, 100 pg, 10 pg, 1pg, 100 fg, 10 fg and 1 fg DNA. PCR products were analyzed using electrophoresis in 1.5% agarose gels in TAE 1 × at 80 V. Gels were stained with 0.5 μg/ml ethidium bromide and photographed under a UV light source.

The PCR amplification products from two positively identified animals to genus and species were sequenced. The sequences were then compared and aligned using the National Center for Biotechnology Information, U.S. National Library of Medicine, Basic Local Alignment Search Tool (BLAST).

## Results

In Table 1, we show the list of collected species with the number of individuals that tested positive for the presence of the parasite *Leishmania* (*L*.) *mexicana* and the number that tested negative. Of the 70 mammal species collected, approximately 1/6 of all species present in Mexico, 24 (34%) had one or more samples that tested positive for the presence of the *Leishmania* (*L*.) *mexicana* parasite. Thirteen species of bats, and one of squirrel, were identified for the first time as *Leishmania* hosts in Mexico. Of the total number of collected individuals (N = 922), 62 tested positive, yielding an average infection rate across all species of 6.7%, although infection rates varied greatly, both temporally and spatially, exhibiting considerable heterogeneity, from 0% to 60%, across distinct collection sites and season of the year. In addition to the percentage that tested positive we also include the 95% confidence interval limits using the Wilson score interval [55,56] for that percentage relative to the null hypothesis that all mammal species had the same baseline infection rate of 6.7%. However, for those species where the number of positives is zero we also calculate the exact probability to obtain this result, (1-p)^N^, where p is the baseline infection rate and N is the number of negative collections.

**Table 1:** List of collected species, ranked by ɛ (see Eq 1 of Methods), with the number of individuals that tested positive for the presence of the parasite *Leishmania* (*L*.) *mexicana* and the number that tested negative.

Species	epsilon	Negative	Positive	Total	Prevalence (%)	95% confidence interval	5% confidence interval	Probability true negative
*Carollia sowelli*	8.83	42	2	44	4.55	18.07	2.28	0.00%
Heteromys gaumeri*	8.80	5	0	5	0.00	46.62	0.59	29.30%
*Peromyscus mexicanus*	8.79	115	6	121	4.96	12.62	3.45	0.00%
Heteromys desmarestianus*	8.72	30	0	30	0.00	50.52	0.50	87.51%
*Molossus rufus*	8.63	1	0	1	0.00	82.00	0.11	6.70%
*Glossophaga soricina*	8.57	9	7	16	43.75	28.92	1.25	0.00%
*Carollia perspicillata*	8.50	8	0	8	0.00	38.49	0.82	42.58%
*Pteronotus parnellii*	8.16	4	0	4	0.00	55.41	0.41	24.22%
*Desmodus rotundus*	8.15	13	1	14	7.14	30.91	1.14	0.00%
*Sturnira lilium*	8.03	58	6	64	9.38	15.59	2.72	0.00%
*Dermanura phaeotis*	8.01	35	1	36	2.78	19.69	2.06	0.00%
*Oryzomys couesi*	7.73	2	0	2	0.00	70.13	0.22	12.95%
Ototylomys phyllotis*	7.56	9	1	10	10.00	36.55	0.89	0.00%
Sigmodon hispidus*	7.28	36	4	40	10.00	18.81	2.18	0.00%
Peromyscus yucatanicus*	7.25	3	0	3	0.00	61.71	0.32	18.78%
*Didelphis virginiana*	7.12	3	0	3	0.00	61.71	0.32	18.78%
*Didelphis marsupialis*	6.44	11	0	11	0.00	28.92	1.25	53.37%
*Philander opossum*	6.25	6	1	7	14.29	43.42	0.67	0.00%
*Centurio senex*	6.01	1	0	1	0.00	82.00	0.11	6.70%
*Artibeus jamaicensis*	5.98	81	5	86	5.81	14.04	3.06	0.00%
*Artibeus lituratus*	5.84	36	3	39	7.69	19.02	2.15	0.00%
*Myotis keaysi*	5.61	2	0	2	0.00	70.13	0.22	12.95%
*Chiroderma villosum*	5.56	5	0	5	0.00	50.52	0.50	29.30%
*Saccopteryx bilineata*	5.30	1	0	1	0.00	82.00	0.11	6.70%
*Sciurus aureogaster*	5.23	71	8	79	10.13	40.75	0.74	42.58%
*Baiomys musculus*	5.21	2	0	2	0.00	70.13	0.22	12.95%
*Artibeus watsoni*	5.13	2	0	2	0.00	70.13	0.22	12.95%
*Choeroniscus godmani*	5.05	10	3	13	23.08	32.07	1.08	0.00%
*Pteronotus personatus*	5.03	3	1	4	25.00	55.41	0.41	0.00%
*Reithrodontomys mexicanus*	4.91	1	0	1	0.00	82.00	0.11	6.70%
*Oryzomys rostratus*	4.87	22	1	23	4.35	24.21	1.59	0.00%
*Micronycteris microtis*	4.23	1	0	1	0.00	82.00	0.11	6.70%
*Oligoryzomys fulvescens*	4.20	6	0	6	0.00	40.75	0.74	34.04%
*Peromyscus leucopus*	3.80	22	4	26	15.38	22.84	1.71	0.00%
*Sturnira ludovici*	3.79	24	1	25	4.00	23.26	1.67	0.00%
*Vampyrodes caraccioli*	3.69	1	0	1	0.00	82.00	0.11	6.70%
*Liomys pictus*	3.61	47	1	48	2.08	17.43	2.38	0.00%
*Glossophaga commissarisi*	3.49	2	6	8	75.00	40.75	0.74	0.00%
*Phyllostomus discolor*	3.48	0	1	1	100.00	82.00	0.11	0.00%
*Lonchorhina aurita*	3.48	1	0	1	0.00	82.00	0.11	6.70%
*Platyrrhinus helleri*	3.36	3	0	3	0.00	61.71	0.32	18.78%
*Uroderma bilobatum*	3.34	4	0	4	0.00	55.41	0.41	24.22%
*Urocyon cinereoargenteus*	2.97	1	0	1	0.00	82.00	0.11	6.70%
*Procyon lotor*	2.95	1	0	1	0.00	82.00	0.11	6.70%
*Myotis velifer*	2.58	4	0	4	0.00	55.41	0.41	24.22%
*Microtus mexicanus*	2.53	16	0	16	0.00	28.06	1.31	67.03%
*Myotis nigricans*	2.47	2	0	2	0.00	70.13	0.22	12.95%
*Leptonycteris curasoae*	2.43	1	1	2	50.00	70.13	0.22	0.00%
*Reithrodontomys fulvescens*	2.08	20	0	20	0.00	21.37	1.86	75.02%
*Neotoma Mexicana*	1.99	5	0	5	0.00	50.52	0.50	29.30%
*Eptesicus fuscus*	1.82	1	0	1	0.00	82.00	0.11	6.70%
*Peromyscus levipes*	1.34	1	0	1	0.00	82.00	0.11	6.70%
*Sorex saussurei*	1.29	3	0	3	0.00	61.71	0.32	18.78%
*Osgoodomys banderanus*	1.21	9	0	9	0.00	34.86	0.95	46.43%
*Liomys irroratus*	1.16	8	0	8	0.00	40.75	0.74	42.58%
*Myotis auriculus*	0.22	2	0	2	0.00	70.13	0.22	12.95%
*Tadaria brasiliensis*	-0.09	1	0	1	0.00	82.00	0.11	6.70%
*Peromyscus hylocetes*	-0.28	2	0	2	0.00	70.13	0.22	12.95%
*Antrozous pallidus*	-0.34	1	0	1	0.00	82.00	0.11	6.70%
*Peromyscus zarhynchus*	-0.46	2	0	2	0.00	70.13	0.22	12.95%
*Chaetodipus hispidus*	-0.71	4	0	4	0.00	55.41	0.41	24.22%
*Peromyscus pectoralis*	-0.73	2	0	2	0.00	70.13	0.22	12.95%
*Neotomodon alstoni*	-0.90	17	0	17	0.00	25.90	1.45	69.24%
*Baiomys taylori*	-1.16	10	3	13	23.08	32.07	1.08	0.00%
*Chaetodipus nelsoni*	-1.24	3	0	3	0.00	61.71	0.32	18.78%
*Neotoma micropus*	-1.27	16	0	16	0.00	28.92	1.25	67.03%
*Peromyscus maniculatus*	-1.37	58	2	60	3.33	15.97	2.64	0.00%
*Peromyscus eremicus*	-1.41	0	1	1	100.00	82.00	0.11	0.00%
*Perognathus flavus*	-1.52	1	0	1	0.00	82.00	0.11	6.70%
*Dipodomys merriami*	-2.01	1	0	1	0.00	82.00	0.11	6.70%

*previously confirmed

**Fig 1:**
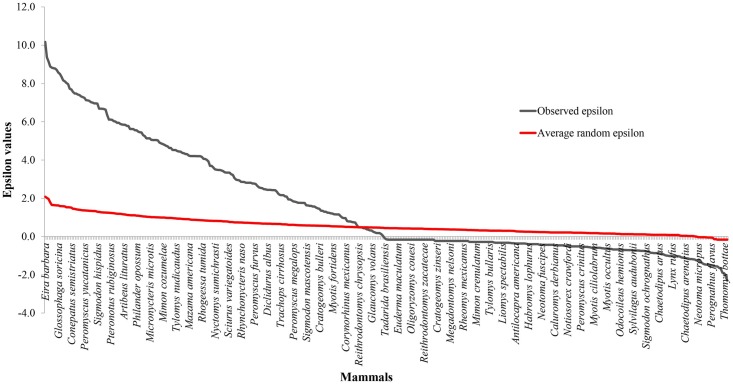
Graph of ranked epsilon values for all mammal species compared to that of a random distribution. The Average random epsilon line represents the distribution of overlaps found by randomly redistributing all collections over those spatial cells that had at least one collection.

Fig 1 shows the ranked values of ε, the statistical measure of degree of co-occurrence used to infer potential biotic interactions (see Eq (1) of the Methods section) for all mammal species as determined from the complex network exhibited in [36] (S1 Table) and considering *Lutzomyia* as a genus. The horizontal axis represents the null hypothesis that sandflies are distributed randomly with respect to mammal distributions, in other words P(B_i_ | I_k_) = P(B_i_) and hence ε = 0. To determine the extent to which collection biases can influence the overall distribution we randomly redistributed all collections over those spatial cells that had at least one collection. This has the effect of removing correlations between one species and another while at the same time preserving any bias associated with under sampling of certain geographical areas. This random re-assortment was repeated 50 times and average values for ε determined for each species.

**Fig 2:**
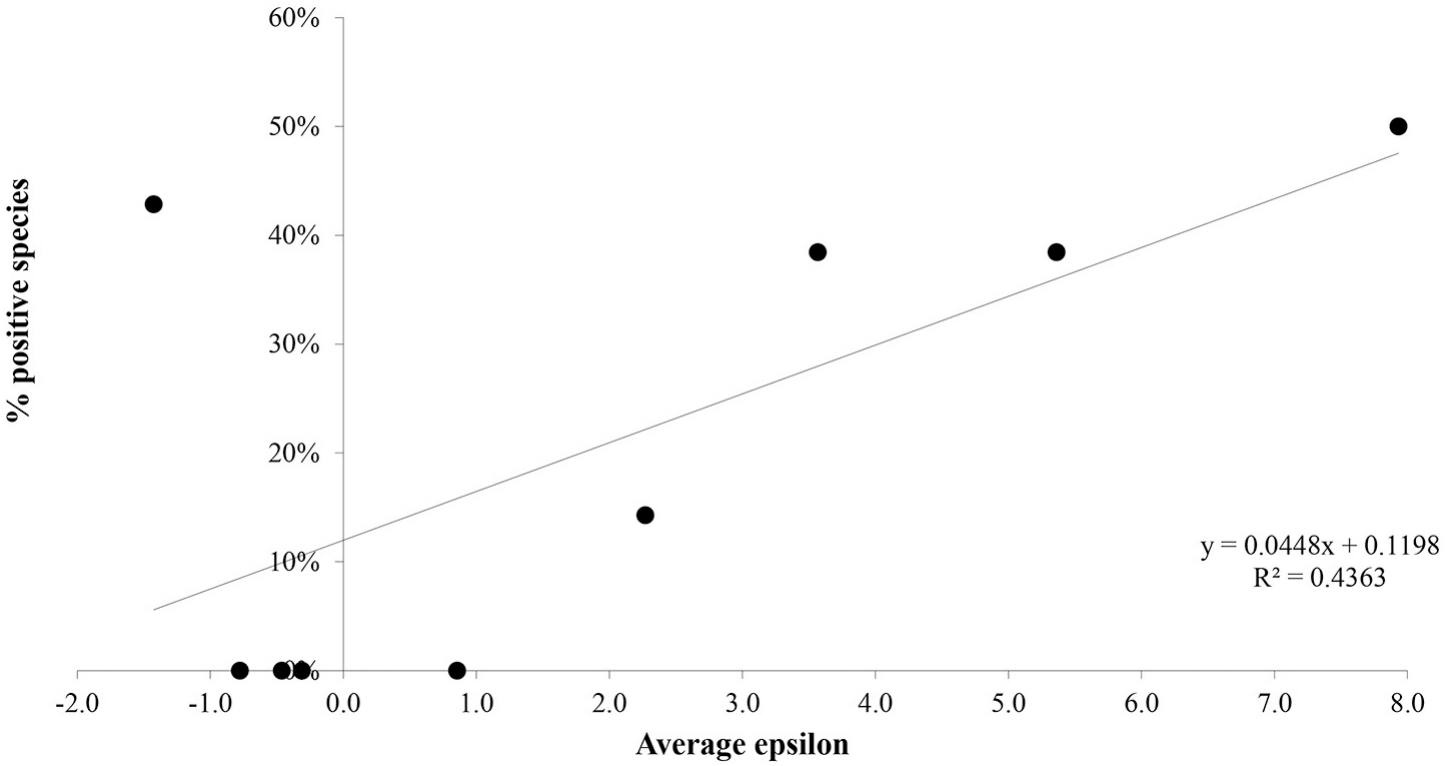
Percentage of species identified as positive for presence of *L*. (*L*.) *mexicana* in relation to mean epsilon value.

**Fig 3:**
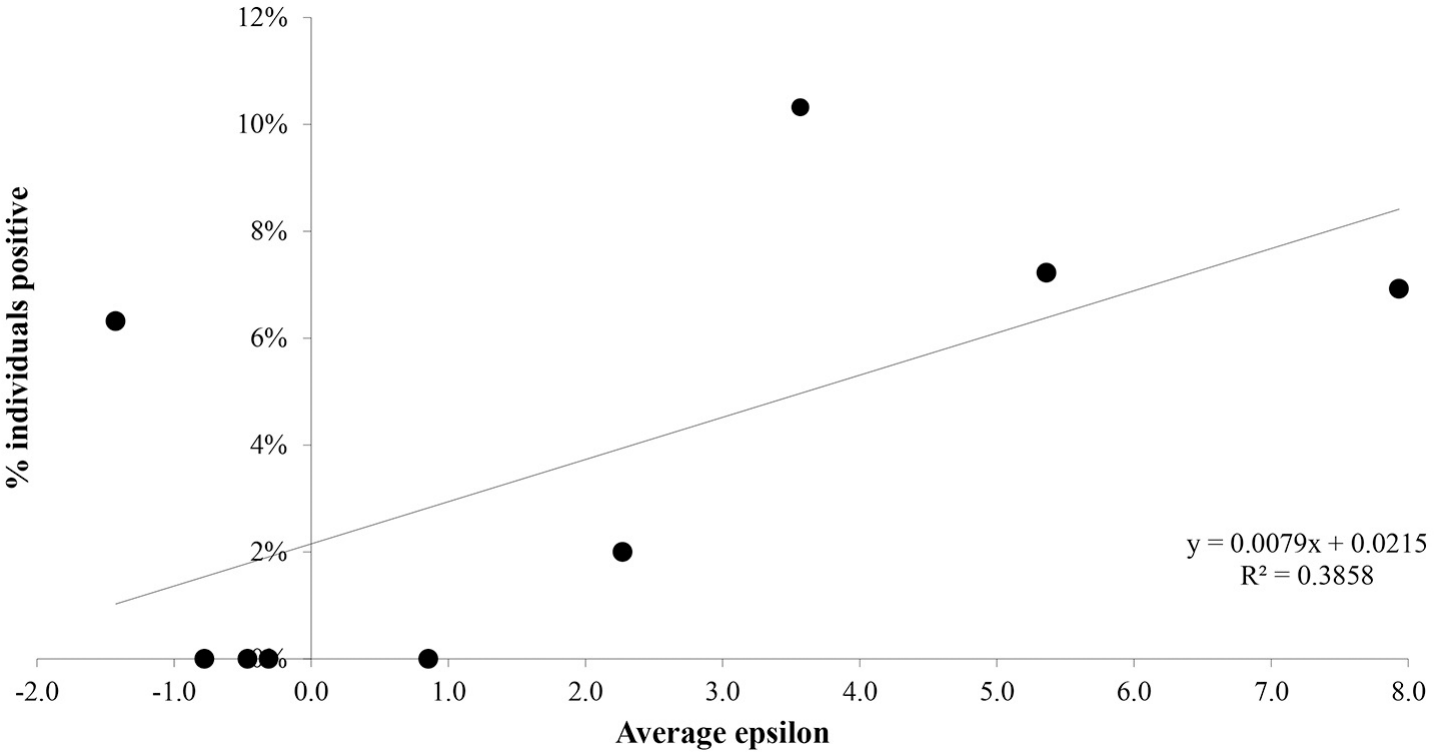
Graph of percentage of individuals identified as positive for presence of *L*. (*L*.) *mexicana* versus mean epsilon value.

To test the predictive power of the risk model, and to better visualize the relationship between ε and the percentage of species that tested positive, we group the list of mammal species ranked by ε into deciles [57] each decile corresponding to 10% of the list, and compute the average value of ε for each decile. The result can be seen in Fig 2. The relative proportion of positives, P, is a strongly increasing function of ε. Note that this regression is only to demonstrate the predictive power of the underlying classification model based on ε, i.e., the statistical significance of co-occurrence. Similarly, in Fig 3 we see the relative correlation between the average value of ε and the percentage of individuals identified as positive. Once again, the relative proportion of positives is a strongly increasing function of ε. Of course, P is a multi-factorial function, P(X_1_, X_2_,…, X_N_) that depends on many factors, such as host competencies. Essentially, here we are considering a regression model for P(X_1_, X_2_,…, X_N_) with respect to the variable X_1_ = ε and ignoring the rest as we do not have the relevant information to include them. Seen as a logistic regression at the species level, the associated relation is: Logit P = -1.415 + 0.186 * ε, with a p value of 0.03 on the regression coefficient. This confirms the statistically significant relation between ε as a statistical measure of geographical overlap and the probability to be a host of *Leishmania (L*.*) Mexicana*.

**Fig 4:**
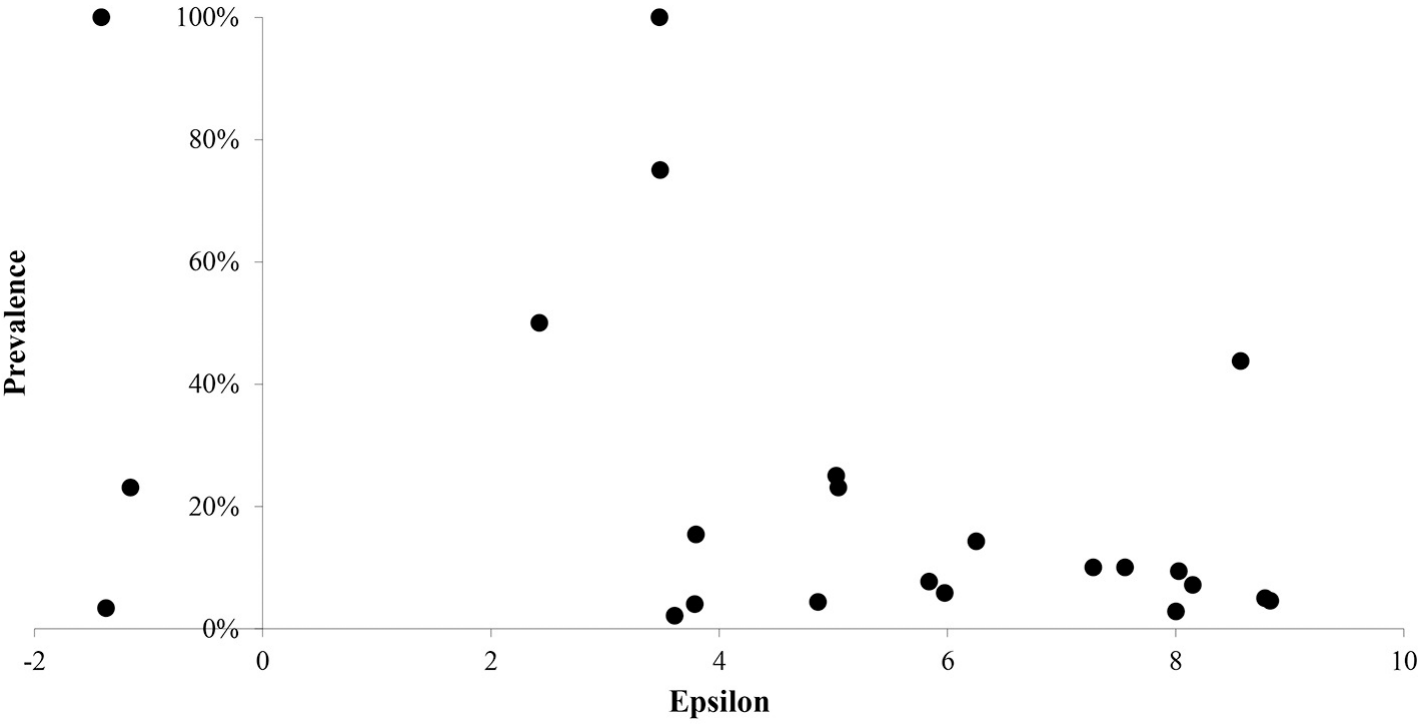
Graph of prevalence versus epsilon by species.

**Fig 5:**
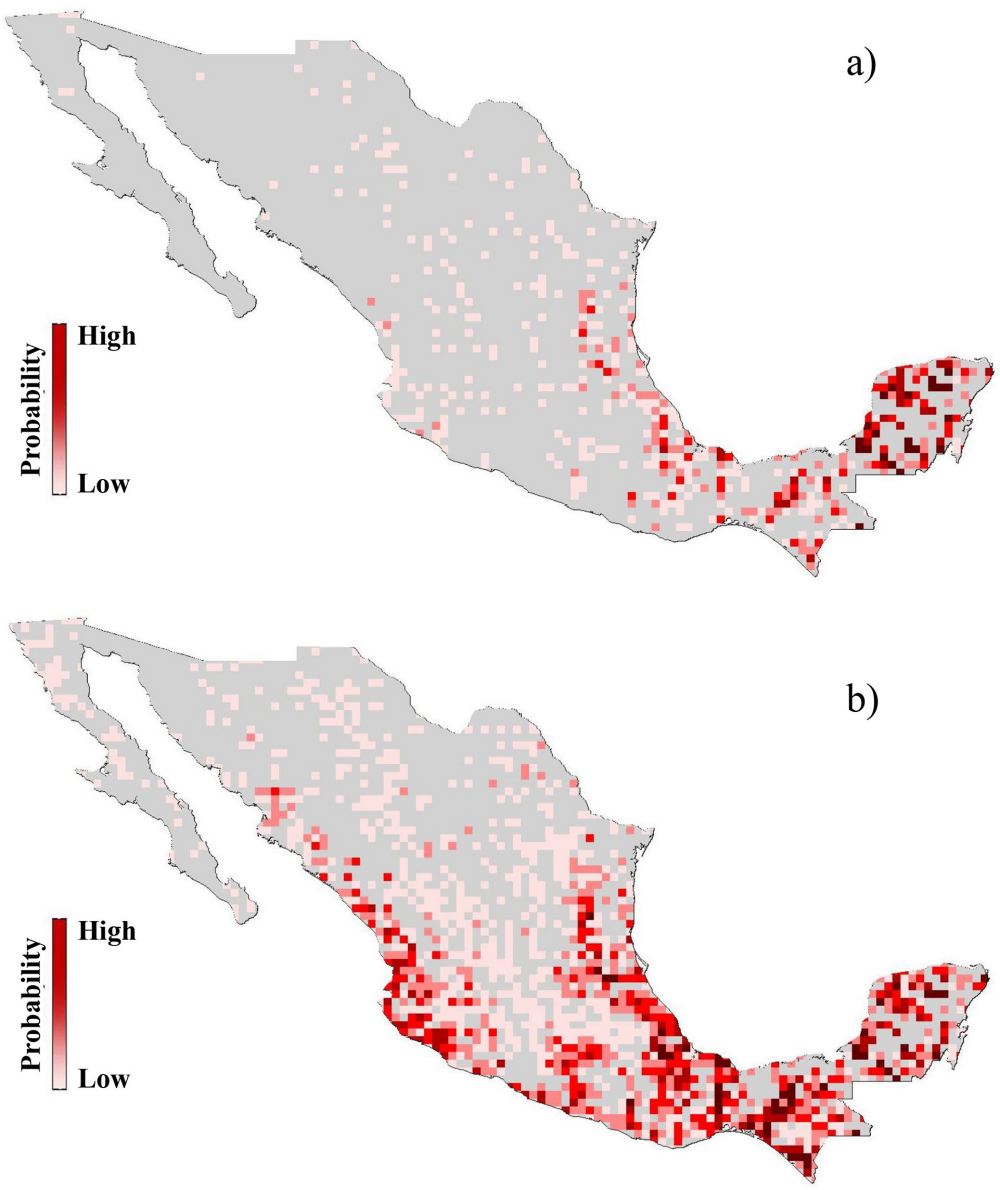
Risk maps of Leishmaniasis: a) determined by using only the 8 previously confirmed hosts, b) determined by using the 22 new confirmed hosts and previously confirmed hosts of *L*. (*L*.) *mexicana*.

In Fig 4 we show a graph of prevalence at the species level versus ε for the 24 positive species, while in Fig 5, we compare the disease risk maps determined by using only the eight previously confirmed hosts of Leishmania versus one using the set resulting from our analysis, where by risk we mean probability of presence of the vector. A clear distinction can be seen between the areas of higher risk between the two models with the present model indicating a much higher degree of risk of presence of *Lutzomyia* in other than the southeast of Mexico.

## Discussion

Host range is an important factor in the dynamics of a zoonosis, both as a variable that affects the overall risk of presence of the disease as well as in terms of determining optimal interventions. An essential factor in determining the importance of a host is its co-distribution with the disease vector. In this paper, we reported the results of an extensive, interdisciplinary, empirical investigation carried out to test the predictions of a model to predict the relative importance of mammal hosts for the pathogen *Leishmania* (*L*.) *mexicana* associated with the emerging disease Leishmaniasis in Mexico. Once again, we emphasise that *importance* here is an “all else being equal” notion, i.e., that the greater the overlap between species the greater the probability of a vector-host interaction and therefore a greater number of infected individuals and a greater probability of transmission. Of course, many other factors—host competence, host abundance etc. will influence the epidemiological importance of a given host.

As the distribution of Fig 1 predicts the most important potential mammal hosts of *Leishmania* (*L*.) *mexicana*, the deviation of the set of real ε values from the two random benchmarks considered, the ε = 0 line and the average random ε curve, shows that the distribution of Lutzomyia is strongly, positively correlated with a large number of mammal species, and, further, that this fact is not explainable by collection biases. Furthermore, the strong asymmetry of the distribution, with 149 potential hosts associated with statistically significant positive correlations with sandflies, compared with only two species with statistically significant negative correlations, is consistent with a hypothesis that sandflies are generalists that are capable of feeding on, and potentially infecting, whatever potential mammal species are available, as has been suggested for several mosquitoes [58]. If sandflies were specialists, associated with a few focal species, one would expect to see high ε values only for those particular species, due to the fact that the focal species are an important and necessary biotic element in the niche of the sandfly, while the other mammals would be incidental and therefore one would expect to see either random association or a positive association mediated by, say, abiotic variables.

The most important results of this paper are in Figs 2 and 3, where we clearly see the significant correlation between the probability for a species or individual to be a host and ε as a measure of the statistical significance of the degree of overlap between vector and host distributions. This is evidence that although many other factors, such as species abundance, species competence etc., enter, the fact that a vector and host must co-occur in order to interact leaves a significant predictive imprint. In other words, the more overlap the more opportunity for host-vector interactions. The multi-factorial nature of the complex relation between host and vector is implicit in that the relation between host probability and ε, although statistically significant, is not characterized by a very high value of R^2^. Especially noteworthy is the decile 1 result, where 3 out of 7 species were identified as hosts—*Baiomys taylori*, *Peromyscus maniculatus* and *Peromyscus eremicus*. These were collected from sites in Jalisco and Nuevo Leon, states from where collections of *Lutzomyia* were previously scarce or non-existent. However, recently, it has been confirmed that various species of *Lutzomyias* are relatively common in these areas. Thus, we believe that a part of the low ranking of these species is due to a systematic bias in the historic collection of *Lutzomyias* towards the southern part of Mexico. The impact of this on the relation between the percentage of positive host species or individuals and ε is substantial. A regression using only the first 9 deciles yields an R^2^ = 0.92 versus 0.44 for all 10 deciles for the species-ε relation, while for the individuals-ε relation the corresponding figures are 0.65 and 0.39. Figs 2 and 3 also illustrate what we mean by host importance. The fact that the percentage of infected individuals decreases as a function of epsilon is consistent with the fact that in the higher deciles (higher ε values) there is a higher probability of a vector encountering an infected host than in the lower deciles (lower ε values). This does not imply, of course, that the pathogen is transmitted. It is however, once again, a necessary if not sufficient condition.

Besides validating both the general methodology as well as the specific model for Leishmaniasis of [36], our results also provide an extensive list of new hosts for *Leishmania* (*L*.) *mexicana* in Mexico that substantially changes what we know about the transmission cycle of the pathogen and the potential efficacy of interventions and/or surveillance efforts. As 33% of collected species tested positive for the presence of *L*. (*L*.) *mexicana* our results are completely consistent with the prediction that *L*. (*L*.) *mexicana* is a very generalist pathogen and that *Lutzomyia* is a very generalist genus. Furthermore, if we consider the probability that a species is a true negative at the 95% confidence level with respect to the null hypothesis of a 6.7% infection rate across all species, then we see that there are no true negatives. The closest is *Heteromys desmarestianus* with N = 30 and a probability of 88% of being a true negative. Interestingly, though, *Heteromys desmarestianus* has previously been identified as a host [49,59]. We also note that, of the 24 identified host species, only four—*Phyllostomus discolor*, *Peromyscus eremicus*, *Glossophaga commissarisi* and *Glossophaga soricina*—are associated with a prevalence that, with 95% confidence according to the Wilson score interval, is greater than the average prevalence across all species. Moreover, the first two species were associated with only one collection so that one would not expect the Wilson score interval to be reliable. The exact probability for an observed 100% prevalence with N = 1 is 6.7%.

Our principle result, as stated previously, is the validation of the methodology and the explicit model of [36] for Leishmaniasis. However, we may also further analyse our experimental results. We have noted that they are quite consistent with the hypothesis that most host species have prevalence values that are compatible with the null hypothesis of a constant prevalence of 6.7% across species. For those positive species with N > 20 in fact prevalence is very stable. Of course, heterogeneities are to be expected. This can be due not only to intrinsic differences in host competence but also, potentially, to spatial heterogeneity associated with epidemiological “hotspots” where many variables together may be favourable for transmission. In Fig 4 we see that there is no noticeable relation between prevalence and ε. We would argue that there should be a dependence if prevalence is averaged for a given species over several geographical locations that systematically sample the range of that species. However, the sampling intensity of the present data is not capable of showing such effects at a species by species level. The fact that the species by species infection rates are consistent with a relatively constant 6.7% prevalence is compatible with the hypothesis that the competence of the different mammal host species is relatively homogeneous. However, as can be seen in Fig 4, the degree of dispersion of the data is large. Partly this is due to the fact that several of the observed prevalences are associated with very small sample sizes. For instance, the species with prevalence of 100% are associated with only one individual. These can be considered as outliers. This heterogeneity in sample size at the species level is one reason why a coarse grained analysis, as observed in Figs 2 and 3, is more appropriate. Taken at face value, the relative homogeneity of prevalence would also argue against any potential dilution effect due to higher biodiversity as this depends on strong competence heterogeneity amongst hosts. In fact, these results would be consistent with the fact that *Lutzomyias* do not differentiate very much among different potential sources of blood meal [60].

The results of this study stand in stark contrast to our previous understanding of the hosts of different species of *Leishmania*: Of the more than 2000 mammal species on the American continent only about 50 (2.5%) have been identified as hosts of *Leishmania* [44], while in Mexico, 8 out of 419 (2.1%) have been identified as hosts [but see 61]. If our result that 33% of collected mammal species are hosts is extrapolated to other non-collected species then potentially hundreds of mammal species could be implicated as hosts of *Leishmania*. Of the collected species that tested positive, 13 were bats, identified for the first time as hosts of *Leishmania* (*L*.) *mexicana* in Mexico [61]. We also identified the grey squirrel as a peri-domestic host species with a high degree of contact with human settlements [62].

If sandflies are generalists, in that they feed off a large number of species across many genera, and pathogen competencies are relatively uniform, as is consistent with our observations, then one would also hypothesize that *Leishmania* (*L*.) *mexicana* is also a generalist in that it can infect a large number of potential host species. In the case of Chagas disease, the impressive genetic plasticity and associated adaptability of *Trypanosoma cruzi* has been amply studied [63–65], as well as its epidemiological implications. Our results suggest that *L*. (*L*.) *mexicana* should also demonstrate a high degree of genetic plasticity and adaptability to be able to infect such a wide array of mammal species. Recent work on the genetics of *Leishmania* seems to be consistent with this viewpoint [66,67].

Given that previously there were only eight confirmed host species of *Leishmania* in Mexico, the results of this paper change the risk landscape for this neglected disease in Mexico, both geographically, and in terms of its control or elimination, with similar potential consequences for other countries. Although it is known that *Lutzomyias* have an ample geographic range and therefore are a risk element for Leishmaniasis in many areas of Mexico, what the present work demonstrates is that there are a large number, and potentially very many more, of hosts involved in the transmission cycle of Leishmaniasis. This complicates both interventions and surveillance. In terms of surveillance we would argue that our model yields a good first approximation as to which host species to survey—those that have been identified as hosts and have the highest ε values. Of course, further field and laboratory work should be carried out to better understand the underlying factors influencing host competence, both at the species level and across different geographical locations for the same species. It is also necessary to test species that are high on the model list but up to now have not been checked. In terms of interventions, with such a large host range, that spans both sylvatic and peri-domestic species, it will be very difficult to eliminate any enzootic transmission cycle, with consequential difficulties in the long term elimination of the vector. Geographically, as can be seen in Fig 5, a risk map derived from the distributions of the eight host species known before the results of this study is completely different to a risk map associated with the 30 species of host that we have now confirmed indicating much higher risk in states such as Jalisco and Nuevo Leon that have until recently been considered low risk compared to the south east.

Our results show that within species collection data there is a great deal of useful information about interspecific interactions and community structure that may be deduced with innovative modelling techniques, such as complex inference networks, and applied to important problem areas such as multi-host diseases. It also shows the importance of the systematic and unbiased collection of data associated with the distributions of potential vectors and potential reservoirs. For instance, the models created showed a higher risk for presence of *Lutzomyia* in the north of Mexico, along the border with the US, than had previously been the case. In fact, recent human cases have been reported in this region, and recent field work has shown that the presence of *Lutzomyia* is more extensive than had been previously thought [44,68].

## Supporting Information

S1 Table Ranked list of mammals according to their epsilon values. (DOCX)

S1 Text Supplementary Field Methodology. (DOCX)
