# Supplementary material for: Can You Judge a Disease Host by the Company It Keeps? Predicting Disease Hosts and Their Relative Importance: A Case Study for Leishmaniasis
Source: PLoS Negl Trop Dis. 2016 Oct 7;10(10):e0005004. doi: 10.1371/journal.pntd.0005004 (PMC5055336; doi:10.1371/journal.pntd.0005004)
Supplement: S1 Text — (DOCX) [file pntd.0005004.s002.docx]

**Supplementary Field Methodology**

Sampling sites were located in 10 Mexican states (Campeche, Chiapas, Estado de México, Jalisco, Michoacán, Nuevo León, Oaxaca, Tabasco, Veracruz y Yucatán; Table 1).

*Animal sampling*

All organisms collected in this study were sampled in strict adherence to all protocols and legislation currently existing in Mexico. SEMARNAT (Mexican Natural Resources Ministry) gave the collection permit in the name of Víctor Sánchez-Cordero (co-author). In addition animal management was in accord with The Animal Care and Use Committee [1].

*Bats*

Bats were caught using 3, 6 and 12 mistnets set in different habitats, such as rivers, vegetation ecotones, croplands, roads and all the places where bat flying trajectories were detected. Mistnets were open from 18:00 to 24:00 hrs. Bat sampling took place from February - 2009 to October 2010, with a total sampling effort of 2,448 nights/mistnet. All individuals collected were taxonomically identifiable and were physically checked for skin lesions.

*Rodents*

Rodents were caught using Sherman traps in transects of different lengths depending of the conditions of the site. As with mistnets, Sherman traps were set in all types of habitat, such as natural vegetation, crop lands etc. Traps were daily baited with vanilla and oat-flakes and kept active from 18:00 to 07:00 hrs the following day. Rodent sampling took place from February - 2009 to October 2010 with a total sampling effort of 5,351 night/traps. Of All individuals caught we took standard measurements and species identification was always possible.

Additionally, we set a number of Tomahawk traps in order to catch some medium-size mammals such as didelphidos. As the objectives of the project required collecting as many species as possible we also collected mammals that were killed in car accidents if they were in sufficiently good condition. These collections comprised less than ca. 3% of total sampling.

We took selected tissues (heart, liver, kidney, lungs, spleen and skin) from all animals collected. All tissues were initially preserved in liquid nitrogen (field) and once in the laboratory they were refrigerated (- 24°C).

Table 1 Localities sampled.

| **Estado** | **Localidad** | **Municipio** |
| --- | --- | --- |
| Campeche | Carretera | Carmen |
| Chiapas | Ixtapangajoya | Ixtacomitán |
| Chiapas | Emiliano Zapata sitio 4 | Ixtacomitán |
| Chiapas | Los Alushes | Palenque |
| Chiapas | MayaBell | Palenque |
| Chiapas | Emiliano Zapata | Ixtacomitán |
| Chiapas | El Limar | Tila |
| Chiapas | Botiojá 1° | Salto del agua |
| Chiapas | Colen-Há | Salto del agua |
| Estado de Mexico | Nevado de Toluca | Zinacantepec |
| Estado de Mexico | La Marquesa | Ocoyoacac |
| Jalisco | Autlan | Autlán de Navarro |
| Jalisco | Chamela | La Huerta |
| Jalisco | Puerto Vallarta | Puerto Vallarta |
| Jalisco | Presa Trijo Mil | Unión de Tula |
| Jalisco | La Taberna | Unión de Tula |
| Jalisco | Coyame Ejido Modelo | Casimiro Castillo |
| Michoacan | Patzingo | San Juan Parangaricutiro |
| Nuevo Leon | Dr. Coss | Doctor Coss |
| Nuevo Leon | Sabinas Hidalgo | Sabinas Hidalgo |
| Nuevo Leon | Bustamante | Bustamante |
| Nuevo Leon | Linares | Linares |
| Nuevo Leon | Rancho San Manuel | Linares |
| Nuevo Leon | Ejido San Nicolas, Predio Colectivo Viejo | Escobedo |
| Nuevo Leon | Cienega de Gonzalez | Santiago |
| Nuevo Leon | Ejido Labores del Ojo | Mina |
| Nuevo Leon | Fraccionamiento Rincon de los Sabinos 2° Sector | Cadereyta Jiménez |
| Oaxaca | Santa Maria Huatulco | Santa María de Huatulco |
| Oaxaca | El Coyul | San Pedro Huamelula |
| Oaxaca | Pochutla | San Pedro Pochutla |
| Oaxaca | Santa Maria Huatulco | Santa María de Huatulco |
| Tabasco | Ejido Morelos Piedra Cunduacan | Cunduacán |
| Tabasco | Poblado C16 Gral. Emiliano Zapata | Cárdenas |
| Tabasco | Grutas de Cocona, Teapa | Teapa |
| Tabasco | Yumka | Centro |
| Tabasco | Paraiso | Paraíso |
| Tabasco | Comalcalco | Comalcalco |
| Tabasco | Villahermosa | Centro |
| Tabasco | Ejido Occidente San Francisco | Paraíso |
| Tabasco | Aquiles serdan, Paraiso | Paraíso |
| Tabasco | Rancheria las flores 2da seccion, Paraiso | Paraíso |
| Tabasco | Cerro, Paraiso | Paraíso |
| Tabasco | Union 1ra. Seccion, Paraiso | Paraíso |
| Tabasco | Libertad 1ra seccion, Paraiso | Paraíso |
| Tabasco | Ra. Emiliano Zapata, Centla | Centla |
| Tabasco | Rancho La Montaña, Jalapita, Centla | Centla |
| Tabasco | Ejido Morelos Piedra Cunduacan 3ra seccion | Cunduacán |
| Tabasco | Teapa | Teapa |
| Veracruz | Los Tuxtlas | Los Tuxtlas |
| Yucatan | Xul | Oxkutzcab |
| Yucatan | Cuncunul | Cuncunul |
| Yucatan | Chichimilá | Chichimilá |

References

1. Sikes R. S & Gannon W. L. The Animal Care and Use Committee of the American Society ofMammalogists Guidelines of the American Society of Mammalogists for the use of wild mammals in research. J. Mammal. 92, 235–253 (2011)
